# Time Course of Lens Epithelial Cell Behavior in Rabbit Eyes following Lens Extraction and Implantation of Intraocular Lens

**DOI:** 10.1155/2021/6659838

**Published:** 2021-01-16

**Authors:** Daijiro Kurosaka, Toshiyasu Imaizumi, Junya Kizawa

**Affiliations:** Department of Ophthalmology, Iwate Medical University School of Medicine, 1-1-1 Idaidori, Yahaba-cho, Morioka, Iwate 0283694, Japan

## Abstract

**Background:**

After cataract surgery, some lens epithelial cells (LECs) transdifferentiate into myofibroblast-like cells, which causes fibric posterior capsule opacification (PCO). Residual LECs differentiate into lens fiber cells, forming Elschnig pearls with PCO. This study was carried out to identify the time course of both types of LEC behavior in rabbit eyes following lens extraction and implantation of an intraocular lens (IOL).

**Methods:**

Phacoemulsification and implantation of posterior chamber IOLs were performed in rabbit eyes. Following enucleation, immunohistochemical methods were used to detect *α*-smooth muscle actin (*α*-SMA), a marker for myofibroblast-like cells, in the pseudophakic rabbit eyes. A mouse monoclonal antibody against *α*-SMA was used.

**Results:**

Soon after the operation, the LECs migrated and covered the lens capsule. Thereafter, the LECs around the anterior capsular margin were always positive for *α*-SMA. However, the distributions of these cells were not consistent. In some specimens, *α*-SMA-positive LECs were present around the IOL optic early after surgery, but most of them had disappeared several weeks after the surgery. The residual cells induced fibrotic PCO. In the other specimens, most LECs around the IOL optic except the anterior capsular margin were negative for *α*-SMA. In the peripheral region covered by the peripheral anterior and posterior capsules, LECs on the posterior capsule always differentiated into lens fiber cells and formed a Soemmering ring. Thereafter, migration of lens fiber cells from the Soemmering ring and differentiation of LECs in situ on the central posterior capsule consisted of Elschnig pearls type of PCO.

**Conclusions:**

Although postoperative LEC behavior is not consistent, residual *α*-SMA-positive LECs induced fibrotic PCO. The lens fiber cells that migrated from the peripheral capsular bag or that were differentiated in situ covered the central posterior capsule, forming Elschnig pearls with PCO.

## 1. Introduction

Residual lens epithelial cells (LECs) migrate and proliferate following cataract surgery [1–24]. These changes produce posterior capsule opacification (PCO), the most common complication of cataract surgery [1–4]. Some LECs that differentiate into myofibroblast-like cells, which can be recognized by the presence of *α*-smooth muscle actin (*α*-SMA), cause fibrotic PCO and contraction of the lens capsule [2–4]. This directly causes visual disturbances [2–4] and postoperative complications such as narrowing of the anterior capsular opening [25, 26]. The other LECs that differentiate into lens fiber cells, which are negative for *α*-SMA, form a Soemmering ring and an Elschnig pearls type of PCO [1, 12].

To understand and reveal LEC behavior after cataract surgery, clinical studies with specular microscopy [5] and investigations using human donor capsular bags with implanted IOL [6–17] and an in vivo animal model [18–23] have been performed. These studies revealed that in the central posterior capsule, a monolayer cellular sheet was first recognized [5, 12]. Then, in some cases, posterior capsular wrinkles appeared with multilayered myofibroblast-like cells (*α*-SMA-positive LECs) [10–12, 16] and bare areas without cells (fibrotic PCO) [11–13]. In other cases, lens fiber cells (*α*-SMA-negative, Elschnig pearls type of PCO) were present on the posterior capsule under IOL optics [1, 8, 11, 12, 20]. However, the detailed process of formation of two types of PCO is not yet fully understood.

To determine the precise time course of the LEC behavior after cataract surgery, it is important to examine how the two types of cells (*α*-SMA positive or negative cells) appear and cause PCO immediately after surgery until the development of PCO. However, it is difficult to obtain human donor capsular bags with implanted IOLs, especially early after cataract surgery. Although there is a possibility that an immunohistochemical study using a pseudophakic animal model can reveal the necessary information, studies have only been performed on specimens obtained 2 weeks after surgery [18]. To determine the precise time course of the LEC behavior after cataract surgery with IOL implantation, we conducted an immunohistochemical study in which we monitored the presence of *α*-SMA in rabbit eyes implanted with IOLs immediately after surgery until the development of PCO.

## 2. Methods

A total of 34 young female Japanese albino rabbits (1.0–1.5 kg) were studied. Each of the following agents was applied topically three times on the day of surgery to one eye of each rabbit: diclofenac sodium, tropicamide, phenylephrine hydrochloride, and nofloxacin. The animals were anesthetized with an intravenous administration of sodium pentobarbital (40 mg/kg) and atropine sulfate (0.02 mg/kg). A superior corneal incision was made with a 3 mm keratome. Continuous curvilinear capsulorhexis of the anterior capsule was then carried out. The lens nucleus was emulsified, and the residual cortex was removed with a phacoemulsifier. The corneal incision was enlarged, and an ophthalmic viscosurgical device (sodium hyaluronate, Johnson and Johnson Vision Care, Inc., New Brunswick, NJ) was injected into the anterior chamber and the lens capsular bag. An IOL (6 mm IOL with 3-piece haptics, Bausch and Lomb, Rochester, NY) was implanted in the bag. After removal of the viscoelastic material, the corneal incision was closed with a continuous 10–0 nylon suture. These procedures adhered to the guidelines of the Association for Research in Vision and Ophthalmology Resolution on the Use of Animals in Research.

The rabbits were humanely killed with an overdose of sodium pentobarbital at 1 d, 3 d, 5 d, 7 d, and 10 d and at 2 wks and 1 mo after the operation. At least two rabbits were studied at each time specified. The eyes were enucleated and immersed in 10% neutral buffered formalin. After fixation, the globe was sectioned at the equator. Specimens were dehydrated through a graded series of alcohols and embedded in paraffin. Tissue sections were then cut from these specimens.

We used a Histostain-SP kit (Invitrogen, Camarillo, CA, USA). The labeled streptavidin-biotin method was used for the immunohistochemical detection of *α*-SMA as described previously [24]. The primary antibody was a mouse monoclonal antibody directed against *α*-SMA (IgG2a, clone 1A4, code no. M851, Dakopatts, Denmark). Peroxidase activity of the secondary antibody was visualized by the addition of a solution containing 3–3'-diaminobenzidine hydrochloride (0.3 mg/ml), 0.005% hydrogen peroxide, and 50 mM TRIS-HCl buffer. The sections were then counterstained with hematoxylin.

Mouse monoclonal IgG2a antibody (clone Dak-G05, code no. X943, Dakopatts) was used as a negative control. No immunoreaction was detected in the negative control. The sphincter and dilator muscles of the iris were used as internal positive controls [27].

## 3. Results

On postoperative day 1, the capsulotomy margin of the anterior capsule was attached to the IOL optic. LECs on the preequatorial anterior capsule became flattened and migrated toward the posterior capsule via the lens equator. These LECs showed negative staining for *α*-SMA (data not shown).

**Figure 1:**
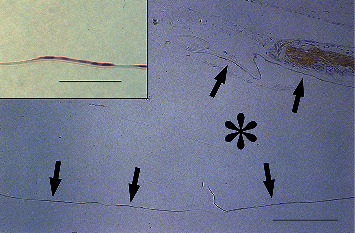
Immunolocalization of *α*-SMA in pseudophakic rabbit eyes 3 days after cataract surgery. A monolayer of LECs covers the inner surface of the anterior and central posterior capsules (arrows). These LECs are negative for *α*-SMA, although the sphincter muscles of the iris are positive for *α*-SMA. bar = 200 *μ*m. The inset shows a higher power view of LECs on the central posterior capsule. Bar = 50 *μ*m. Sections were stained with 3-3'-diaminobenzidine hydrochloride and counterstained with hematoxylin. The asterisk indicates the IOL optic.

By postoperative day 3, a monolayer of LECs covered the inner surface of the central posterior capsule (Figure 1). These LECs were also negative for *α*-SMA.

**Figure 2:**
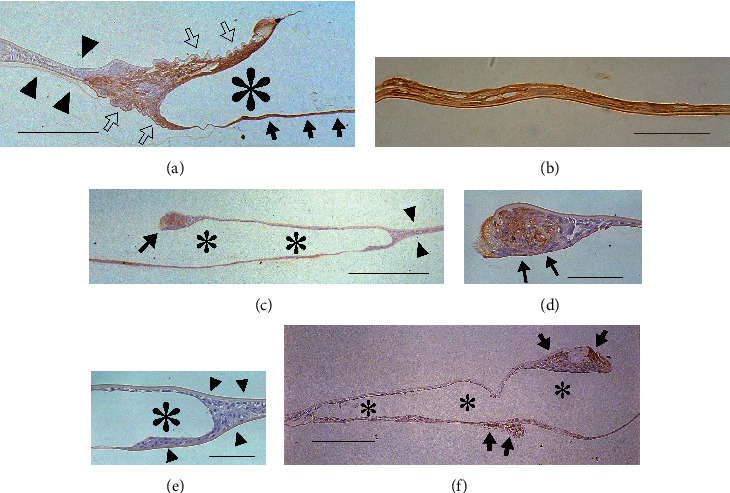
Immunolocalization of *α*-SMA in pseudophakic rabbit eyes 5 days after cataract surgery. The flattened and multilayered LECs are positive for *α*-SMA. The specimens could be divided into two groups according to the distribution of these *α*-SMA-positive LECs around the IOL optics. (a) Surrounding type: LECs positive for *α*-SMA surround the IOL optic. The anterior and posterior capsules around such LECs appear wrinkled (open arrows) except for the posterior capsule under the IOL optic (arrows). The inner surface of the peripheral anterior and posterior capsule is covered by a monolayer of LECs, with these two surfaces being attached to each other like a zipper (arrowheads), which are separated from the IOL optic by *α*-SMA-positive LECs around the IOL optic edge. These LECs are negative for *α*-SMA. Bar = 250 *μ*m. (b) Higher power view of (a): the central posterior capsule under the IOL optic is covered by *α*-SMA-positive LECs without wrinkling of the capsule. (c) Restricted type: LECs positive for *α*-SMA are present only around the margin of the anterior capsule (arrow). Other LECs around the IOL optics are negative for *α*-SMA, with most of them forming a monolayer. The inner surface of the peripheral anterior and posterior capsule is also covered by a monolayer of *α*-SMA-negative LECs (arrowheads). Bar = 400 *μ*m. (d) Higher power view of (c): the margin of the anterior capsule is covered with *α*-SMA-positive, flattened LECs (arrows). Bar = 75 *μ*m. (e); higher power view of (c): LECs around the edge of the IOL optic and on the inner surface of the peripheral anterior and posterior capsules are *α*-SMA-negative (arrowheads). Bar = 75 *μ*m. (f) Restricted type: LECs positive for *α*-SMA are present around the margin of the anterior capsule and on small areas of the posterior capsule (arrows). Bar = 200 *μ*m. (a–f) Sections were stained with 3-3'-diaminobenzidine hydrochloride and counterstained with hematoxylin. The asterisks indicate the IOL optic.

On postoperative day 5, the flattened and multilayered LECs were positive for *α*-SMA. LECs around the capsulotomy margin of the anterior capsule were always *α*-SMA-positive (Figures 2(a), 2(c), 2(d), 2(f)). On the other hand, in the peripheral space without IOL optics, the inner surfaces of the peripheral anterior and posterior capsules were always covered by a monolayer of LECs. These peripheral capsules were attached to each other like a zipper, with the associated LECs staining negative for *α*-SMA (Figures 2(a) and 2(c)).

However, the specimens could be divided into two groups according to the distribution pattern of the *α*-SMA-positive LECs around the IOL optics, which is consistent with a previous study by Matsushima et al. [18]. In the first group, the IOL optic was surrounded by *α*-SMA-positive LECs (“surrounding type”; Figures 2(a) and 2(b)). In the other group having a restricted type of distribution, the *α*-SMA-positive LECs were almost only present around the margin of the anterior capsule (“restricted type”; Figures 2(c) and 2(f)). Except for the margin of the anterior capsule, the anterior and posterior capsules were mostly covered by LECs that typically formed a monolayer (Figures 2(c) and 2(e)). Alpha-SMA-positive LECs were sometimes detected on the restricted area of the central posterior capsule (Figure 2(f)). The results on postoperative day 7 resembled those on postoperative day 5.

**Figure 3:**
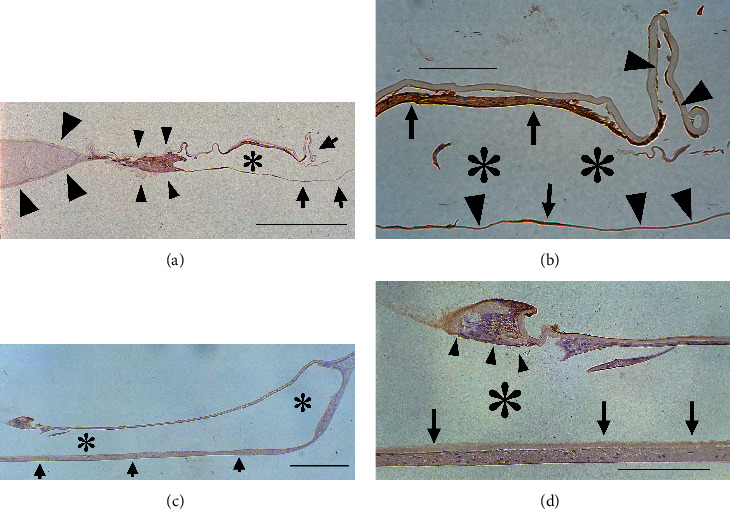
Immunolocalization of *α*-SMA in pseudophakic rabbit eyes 10 days after cataract surgery. (a) Surrounding type: The flattened and *α*-SMA-positive LECs cover some parts of the anterior and posterior capsules around the IOL optics. The other parts of the anterior and posterior capsules around the IOL optics have no LECs (arrows). The LECs on the peripheral posterior capsule begin to differentiate and form the Soemmering ring and are negative for *α*-SMA (large arrowheads), which are separated from the IOL optic by *α*-SMA-positive LECs around the IOL optic edge (small arrowheads). Bar = 250 *μ*m. (b) Higher power view of (a): although there are some *α*-SMA-positive LECs on the anterior and posterior capsules (arrows), LECs are absent from other areas of these capsules (arrowheads). Bar = 50 *μ*m. (c) Restricted type: the LECs on the posterior capsules begin to differentiate into lens fiber cells (arrows). Bar = 400 *μ*m. (d) Higner power view of (b): the margin of the anterior capsule is covered with *α*-SMA-positive, flattened LECs (arrowheads), whereas LECs on the posterior capsule begin to differentiate into lens fibers (arrows). Bar = 125 *μ*m. (a–d) Sections were stained with 3-3'-diaminobenzidine hydrochloride and counterstained with hematoxylin. Asterisks indicate the IOL optic.

On postoperative day 10, the LECs around the capsulotomy margin of the anterior capsule were always *α*-SMA-positive as were the specimens on postoperative day 5. In the peripheral space without IOL optics, *α*-SMA-negative LECs on the inner surfaces of the posterior capsules began to differentiate into lens fiber cells (Figures 3(a), 3(c), and 3(d)) to form Soemmering ring (Figure 3(a)). The distribution of *α*-SMA-positive LECs around the anterior capsular margin did not change in the restricted type but did in the surrounding type: these LECs were absent on some of the anterior and posterior capsules surrounding the IOL optics (Figures 3(a) and 3(b)).

**Figure 4:**
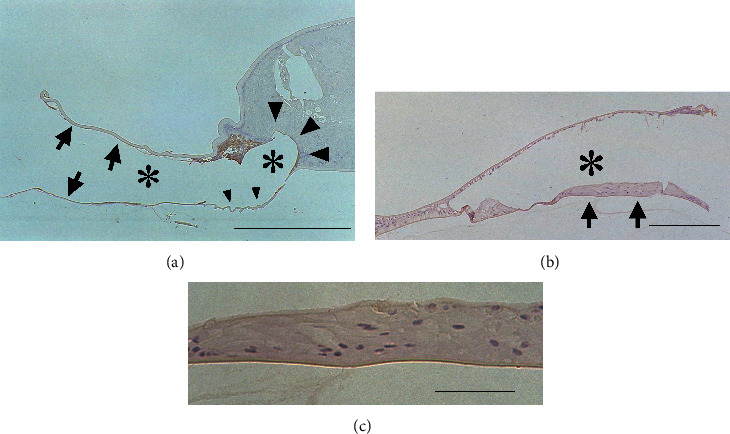
Immunolocalization of *α*-SMA in pseudophakic rabbit eyes 14 days after cataract surgery. (a) Surrounding type: most of the capsules around the IOL optics are devoid of LECs (arrows). Some posterior capsules are wrinkled without LECs (small arrowheads). Some *α*-SMA-positive LECs are present on the capsules, and the Soemmering ring has increased in size. The separation between the IOL optic and the Soemmering ring is lost. Part of the Soemmering ring has begun to attach to the IOL optic (large arrowheads). Bar = 200 *μ*m. (b) Restricted type: the differentiation of LECs under the IOL optic has progressed to form the Elschnig pearls type of opacification (arrows). Bar = 200 *μ*m. (c) Higher power view of (b): the differentiated lens fiber cells are recognizable. Bar = 50 *μ*m. (a–c) Sections were stained with 3-3'-diaminobenzidine hydrochloride and counterstained with hematoxylin. Asterisks indicate the IOL optic.

On postoperative day 14, in the surrounding type of distribution (Figure 4(a)), the separation between the IOL optic and Soemmering ring was lost. The size of the Soemmering ring increased, and some lens fiber cells within the Soemmering ring began to attach to the IOL optic. The cell-free area on the anterior and posterior capsules spread. The area on the posterior capsule attached to the *α*-SMA-positive LECs decreased. Some posterior capsules were wrinkled without LECs.

In the restricted type of distribution pattern (Figures 4(b) and 4(c)), the differentiation of LECs into lens fiber cells under the IOL optic progressed to form the Elschnig pearls type of opacification.

**Figure 5:**
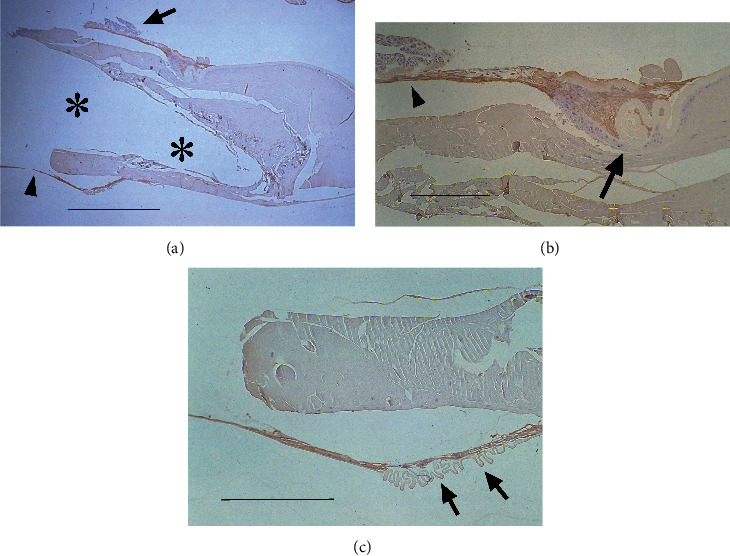
Immunolocalization of *α*-SMA in pseudophakic rabbit eyes 1 month after cataract surgery. (a) The capsular bag is filled with lens fibers. Differentiated LECs are also present between the anterior surface of the IOL optic and the anterior capsule. Some of these lens fibers migrated beyond the anterior capsule margin and formed posterior synechia to the iris (arrow). The lens fibers migrated onto the posterior capsule from the periphery to the center (arrowhead), which showed no LECs. Bar = 800 *μ*m. (b) Higher power view of LECs around the margin of the anterior capsule (arrow). The arrowhead indicates the posterior synechia of the lens fibers to the iris. Bar = 250 *μ*m. (c) Higher power view of lens fibers on the central posterior capsule. Differentiated LECs, which form the Elschnig pearls type of opacification, are present over the *α*-SMA-positive LECs attached to the wrinkled posterior capsule (arrows). Bar = 250 *μ*m. (a–c) Sections were stained with 3-3'-diaminobenzidine hydrochloride and counterstained with hematoxylin. The asterisks indicate the IOL optic.

One month after surgery, it became difficult to divide the specimens according to the distribution pattern of *α*-SMA-positive LECs because these LECs existed only in limited areas, such as the anterior capsular margin and some parts of the posterior capsule. Some areas of the anterior and posterior capsules surrounding the IOL optic without LECs, which had been observed on postoperative day 14, decreased. The LECs that covered the inner surface of the anterior capsule exhibited a normal cuboidal appearance. The peripheral capsular bag was filled with lens fiber cells and had formed a Soemmering ring. Lens fiber cells were present at the posterior capsule under the IOL optic and had formed Elschnig pearls type of opacification. Lens fiber cells were occasionally also present between the anterior surface of the IOL optic and the anterior capsule. Some of these lens fiber cells migrated beyond the anterior capsular margin and formed posterior synechia to the iris (Figures 5(a) and 5(b)). These LECs and lens fiber cells were negative for *α*-SMA.

When the central posterior capsule was completely covered by the lens fiber cells, i.e., the Elschnig pearls type of opacification, we could not ascertain whether these lens fiber cells resulted from the differentiation of the LECs on the posterior capsule in situ or whether they had migrated from the peripheral portion of the lens capsular bag, namely Soemmering ring. However, in some specimens, the lens fiber cells were over residual *α*-SMA-positive LECs, which formed fibrotic PCO accompanied by wrinkling of the posterior capsule (Figure 5(c)). This observation implies that the LECs differentiated into lens fiber cells at the peripheral capsular bag and that these fiber cells migrated to the central posterior capsule and formed the Elschnig pearls type of opacification.

The time course of the behavior of *α*-SMA-positive and negative LECs is summarized in Table 1.

## 4. Discussion

The present study demonstrated that undifferentiated LECs migrated and covered the lens capsule soon after IOL implantation. Some LECs subsequently differentiated into *α*-SMA-positive LECs by the fifth postoperative day. LECs at the anterior capsular margin were always positive for *α*-SMA, but those around the IOL optics were occasionally positive and they decreased within 2 weeks of the operation. On the other hand, in the peripheral space without IOL optics, the inner surface of the peripheral anterior and posterior capsules was always covered by a monolayer of LECs. Those on the peripheral posterior capsule differentiated into lens fiber cells and formed a Soemmering ring. On the other hand, the Elschnig pearls type of opacification on the central posterior capsule consisted of lens fiber cells migrating from the Soemmering ring and/or the differentiation of LECs in situ.

In this study, *α*-SMA-positive LECs appeared 5 days after surgery. The specimens from 5 days to 2 weeks after surgery were divided into two groups according to the LEC distribution. However, since the number of these LECs then decreased, it became difficult to group the specimens one month after surgery.

Matsushima et al. [18] also reported that specimens of rabbit pseudophakic eyes 2 weeks after cataract surgery could be divided into two groups depending on whether the *α*-SMA-positive LECs separated the IOL optic edge from the peripheral lens fiber cells. These findings were consistent with our results. Moreover, they reported that the two groups were almost evenly distributed. Uusitalo and Kivelä [17] reported the distributions of the *α*-SMA-positive LECs were not unique to human pseudophakic eyes obtained at autopsy. Ness et al. [9] also found two types of histopathologic sections of human cadaver eyes. One had lens fiber cells within the Soemmering ring that were separated from the IOL optics by flattened LECs, the same as our “surrounding type,” and the other showed lens fiber cells attached directly to the IOL optics, which resembles our “restricted type.” However, the period from surgery to enucleation was unknown. We found it became difficult to divide the specimens according to the distribution pattern of *α*-SMA-positive LECs one month after surgery because these LECs existed only in limited areas, such as the anterior capsular margin in both types. Therefore, there is a possibility that the latter showed changes obtained late after surgery.

It is unclear how the distribution of *α*-SMA-positive LECs becomes divided into two groups. Matsushima et al. [18] reported that IOL materials did not affect it. In aphakia eyes after cataract surgery, *α*-SMA-positive LECs were also present around the anterior capsular margin [24]. Tan et al. [28] reported that the presence of *α*-SMA-positive LECs was dependent on the size of the anterior capsulorhexis: *α*-SMA-positive LECs were not observed with a 2.0 mm diameter anterior capsulorhexis but were observed with a 4.0 or 6.0 mm diameter. There is a possibility that the size of the anterior capsulorhexis might affect the distribution of *α*-SMA-positive LECs.

On the other hand, the LECs around the anterior capsular margin were always positive for *α*-SMA after the fifth postoperative day. In human cadaver eyes, the LECs around the anterior capsular margin were always positive for *α*-SMA [11, 12]. Transforming growth factor-*β* (TGF-*β*), which is present in the aqueous and vitreous humor [29–32], induces the myodifferentiation of LECs in vitro and in vivo [2–4, 33–35]. An anterior capsular margin is a place that comes into direct contact with the aqueous humor. TGF-*β* in the aqueous humor may influence the myodifferentiation of LECs around the anterior capsular margin.

In this study, most *α*-SMA-positive LECs around IOL optics except for the anterior capsular margin were diminished, which formed a cell-free area on the capsule around the IOL. In an immunohistochemical examination using a whole mount of a human donor capsular bag implanted with an IOL, the central posterior capsules were largely free of LECs, and in the other area, there were *α*-SMA-positive LECs that detached from the wrinkled areas around the optic edge [12] and showed cell degeneration [11]. In anterior capsular opacification around the IOL optic of monkey eyes, flattened LECs were initially abundantly observed, but degeneration and cellular debris were observed 2 months after the operation [21].

The migration of LECs in humans starts within days after cataract surgery, with the disappearance of LECs occurring between 30 and 90 days postoperatively in 87% of patients with implantation of polyacrylic IOLs, 8% with silicone IOLs, and 15% with PMMA IOLs [36]. These findings suggest that the cell-free area appeared due to cell regression after the LECs had covered it. We previously reported that the disappearance of myofibroblast-like LECs occurred at the margin of the anterior capsule in aphakic rabbit eyes due in part to apoptosis [37]. In human eyes, LECs undergo apoptosis in the early phase after cataract surgery [22]. Although further studies are required, there is a possibility that the formation of a cell-free area is related to cell death.

In this study, in the peripheral space without IOL optics, the inner surfaces of the peripheral anterior and posterior capsules were covered by a monolayer of LECs 5 days after cataract surgery. Then, LECs on the posterior capsule differentiated into lens fiber cells and formed a Soemmering ring as previously reported [6, 12]. After that, we observed differentiated lens fibers not only between the IOL optic and the anterior capsule but also over the residual *α*-SMA-positive LECs, which means that differentiated lens fibers can migrate onto the central posterior capsule from the peripheral capsular bag. They induced the formation of the Elschnig pearls type of PCO. Even if the Soemmering ring had been separated from the central posterior capsule by *α*-SMA-positive LECs, this separation was broken by the increase in the size of the Soemmering ring.

These findings are consistent with previous studies with human pseudophakic eyes. Volk et al. [38] showed reopening of the anterior and posterior capsular fusion at the IOL optic edge when lens fiber cells from the Soemmering ring pushed out, and they migrated on the IOL optic under the anterior capsule but also on the posterior capsule behind the IOL optic, as shown by optical coherence tomography. On the other hand, it was previously reported that the Elschnig pearls type of PCO is produced by the differentiation of LECs into lens fibers on the posterior capsule in situ [23, 39]. Such differentiation occurred in the present study in the case of the restricted type. Therefore, the formation of the Elschnig pearls type of PCO, which indicates the presence of lens fibers between the posterior capsule and the IOL optic, may come from not only the differentiation of LECs in situ but also migration from the peripheral capsular bag, namely the Soemmering ring.

## 5. Conclusions

The present study showed that the behaviors of the *α*-SMA-positive LECs were not consistent, and some of them disappeared, forming cell-free areas on the posterior capsule. On the other hand, *α*-SMA-negative LECs formed the Elschnig pearls type of PCO by both differentiation on the central posterior capsule under the IOL optic and also by migration from the Soemmering ring. Although further investigations are required to clarify the mechanism of these phenomena, these findings may lead to the prevention of PCO.

## Figures and Tables

**Table 1:** Immunohistological results of specimens.

		Time since surgery					
		3 days		5 days	7–10 days	14 days	1 month
Around IOL optic	Anterior capsular margin	N	R	P	P	P	P, LF
			S	P	P	P	
	Anterior capsule	N	R	N	N	N	N, LF
			S	P	P, CF	P, CF	
	Cental posterior capsule	N	R	N	N (LF)	LF	P, LF
			S	P	P, CF	P, CF	
	Around optic edge	N	R	N	N, LF	N, LF	LF
			S	P	P	P, LF	

Peripheral capsular bag	Anterior capsule	N	R	N	N	N	N
			S	N	N	N	
	Posterior capsule	N	R	N	N (LF)	LF	LF
			S	N	N (LF)	LF	

R: restricted type, S: surrounding type, N: *α*-SMA-negative LECs, P: *α*-SMA-positive LECs, LF: lens fiber cells, CF: cell free area.

## Data Availability

All relevant data are included within the article and are available from the corresponding author upon request.
